# Development of an AI-powered AR glasses system for real-time first aid guidance in emergency situations

**DOI:** 10.1186/s13040-025-00473-6

**Published:** 2025-08-26

**Authors:** Mohammed Abo-Zahhad, Mostafa N. Zakaria, Farida M. Sharaf, May M. Ismaiel, Habiba Hafrag, Yousef M. Amer

**Affiliations:** 1 Department of Electronics and Communications Engineering, Egypt-Japan University of Science and Technology, New Borg El-Arab City, Alexandria Egypt; 2 Department of Electrical and Electronics Engineering, Assiut University, Assiut, Egypt

**Keywords:** First aid emergency assistance, AI-Powered, Augmented reality, Jetson applications

## Abstract

AI-powered augmented reality (AR) systems provide real-time, hands-free guidance, enabling untrained individuals to respond effectively in emergencies. By combining AI decision-making with AR visuals, they enhance awareness, reduce stress, and help bridge the gap before professional help arrives, especially in critical or underserved settings. This paper introduces an AI-powered augmented reality (AIAR) platform designed to assist untrained bystanders in delivering effective emergency response. By combining real-time object detection (YOLOv5), edge computing (Jetson Nano), and multimodal guidance through AR overlays and text-to-speech, the system targets four critical scenarios—bleeding, burns, fainting, and CPR—using specialized detection models built on domain-specific datasets. The decision-making and instructional flows align with medically validated protocols to ensure accuracy and relevance. Testing demonstrated the system’s high usability and responsiveness in delivering hands-free, context-aware guidance, though limitations related to hardware, model performance, and real-world conditions remain. Future developments include more compact AI hardware, wearable AR enhancements, adaptive interaction, clinical trials, and the integration of advanced AI, such as generative models and natural language interaction.

## Introduction

### Augmented reality and artificial intelligence in healthcare

**Fig. 1:**
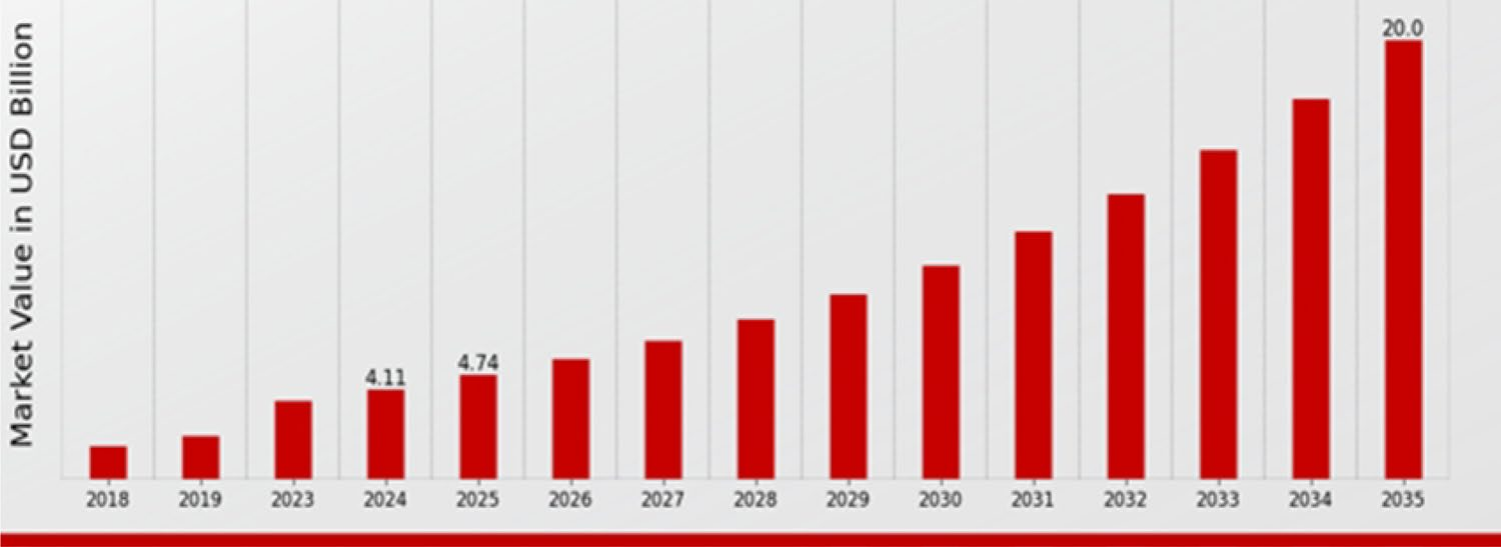
Augmented reality in healthcare market growth, trends [4]

In recent years, augmented reality (AR) and artificial intelligence (AI) have rapidly advanced healthcare by making clinical systems more intelligent, accessible, and responsive. AR enriches the real-world environment with real-time digital overlays, such as images, text, or 3D models. This supports precision and speed in applications like surgery, rehabilitation, training, and emergency care. AI brings data-driven intelligence, enabling machines to learn, recognize patterns, and make clinical decisions, particularly through machine learning and deep learning. When combined, AR and AI create robust, context-aware systems that enhance diagnostics, medical education, and real-time emergency response. These technologies enable surgical navigation, interactive training with instant feedback, and AI-powered AR glasses that guide untrained users in delivering life-saving first aid. With the evolution of lightweight AR hardware and advanced AI models like convolutional neural networks (CNNs), their impact on real-world healthcare continues to grow, improving outcomes and expanding access to critical medical support [1–3]. Figure 1 illustrates the augmented reality in healthcare market growth trends [4]. AR and AI technologies are increasingly adopted across diverse healthcare fields, including diagnostics, surgery, training, and rehabilitation. Their global demand continues to grow, driven by the urgent need to reduce preventable deaths, particularly in emergencies where rapid response is vital. The World Health Organization (WHO) reports that 1.5 million deaths annually result from injuries where timely, effective intervention could save lives [5].

**Table 1:** Applications of AR and AI in healthcare

Application	Technology	Benefits	Examples
Surgical Navigation	AR + AI	Improved precision, reduced errors	Overlaying MRI data during surgery
Medical Training	AR + AI	Realistic simulations, adaptive feedback	AR-based CPR training
Emergency First Aid	AR + AI	Real-time guidance, accessibility	AI-driven burn detection via AR glasses
Diagnostics	AI	Faster, accurate analysis	AI-powered radiology

In many cases, inadequate or delayed first aid leads to severe complications or fatalities. As highlighted in Table 1, AR and AI offer a promising solution by empowering untrained individuals to respond quickly and correctly under the guidance of intelligent systems. Despite their transformative potential, the widespread adoption of AR and AI in healthcare faces significant challenges, including high implementation costs, ethical concerns around data privacy, and limited digital literacy among medical staff, particularly in low- and middle-income countries with unreliable infrastructure [6]. However, future trends suggest continued growth. Advances in edge AI will enable real-time diagnostics through local processing on wearable or bedside devices, while lighter, more affordable AR headsets are becoming increasingly accessible. Emerging technologies like digital twins and generative AI are also paving the way for personalized treatment simulations, signaling a shift toward smarter, more responsive, and decentralized healthcare delivery models.

In this paper, the integration of AR and AI for emergency first aid guidance forms the foundation of the proposed system. The goal is to develop a wearable solution capable of detecting emergencies and delivering immediate, step-by-step instructions through AR visuals, supported by AI-driven decision-making. While AR and AI have shown strong potential in clinical and training settings, their application in real-world, non-clinical emergency scenarios remains limited. The following section examines the key barriers to effective first aid in real-world environments.

### Barriers, limitations of the first aid gap

The importance of timely and accurate first aid in emergencies—such as bleeding, burns, fainting, and cardiac arrest—is critical, especially in out-of-hospital settings where professional medical help may be delayed. However, numerous barriers hinder an effective response. Studies show that fewer than 10% of cardiac arrest victims receive timely Cardiopulmonary resuscitation (CPR), largely due to a lack of awareness, confidence, and practical preparedness among bystanders [7]. Even trained individuals often fail to recall procedures under stress, while traditional training methods offer limited real-world reinforcement [8]. Technology-based solutions like apps and telehealth lack the hands-free, context-aware functionality needed in high-pressure situations [9]. Diagnostic uncertainty, geographic and socioeconomic disparities, and psychological barriers such as fear of error or legal consequences further complicate the issue [10]. Current training models—whether face-to-face, online, or VR—each have significant limitations in accessibility, effectiveness, or scalability [11]. In response, this paper proposes an AI-powered, wearable AR system capable of detecting emergencies like bleeding, burns, fainting, and cardiac arrest, and providing real-time, step-by-step visual and audio guidance. Designed to empower untrained bystanders, the system addresses critical gaps in traditional first aid delivery by offering an intelligent, context-aware, and hands-free intervention method. Table 2 presents a comparative analysis of conventional and technology-assisted first aid approaches, emphasizing the benefits of AI/AR integration in emergency care [12].

The motivation behind this research stems from the urgent need to simplify emergency medical response during the critical minutes before professional help arrives. While previous sections have highlighted the transformative potential of AR and AI in healthcare and the shortcomings of current first aid practices, there remains a lack of practical, scalable solutions that enable untrained bystanders to respond effectively in real time. This system aims to fill that gap by leveraging wearable technologies to shift emergency response from institutions to individuals. It focuses on translating complex medical protocols into intuitive, context-aware instructions using intelligent systems.

The key contributions of the paper are to present a novel integration of real-time artificial intelligence (AI) and augmented reality (AR) to enhance amateur first aid response during emergencies. Thee include:

- Development of a context-aware AR guidance system using YOLOv5-based object detection embedded in AR glasses to identify bleeding, burns, fainting, and cardiac arrest, emergency conditions, and deliver tailored visual and auditory instructions.
- Hands-free AI-AR interface implementation via Jetson Nano, Raspberry Pi V2 camera, and GPU acceleration for real-time, edge-based deployment in low-resource or out-of-hospital settings.
- Creation of domain-specific annotated datasets for training deep learning models to recognize first aid scenarios, including injury severity and the presence of medical supplies.
- Incorporation of multimodal communication (speech, text, and visual symbols) to improve accessibility, particularly for users with low digital literacy in high-stress situations.

**Table 2:** Comparison of first aid delivery methods

Method	Accessibility	Real-Time Guidance	Hands-Free	Limitations
Manual Training	Low	No	No	Static, infrequent practice
Mobile Apps	Moderate	Limited	No	Requires input, independent of users or devices
Telehealth	Moderate	Yes	No	Dependent on connectivity
AR/AI System	High	Yes	Yes	Initial cost and training needed

## Literature review

AI is revolutionizing emergency healthcare by enhancing triage, clinical decision-making, and patient monitoring. As discussed by Kachman et al. (2024), AI technologies—including machine learning, deep learning, and generative AI—improve accuracy in self-triage through advanced chatbots, outperforming traditional symptom checkers. In emergency departments (EDs), AI assists with triage level assignment, automated documentation (e.g., Nuance DAX), differential diagnosis, and cardiac risk stratification. It also supports medical imaging for detecting fractures or hemorrhages and employs monitoring tools like eCART to predict critical deterioration, such as sepsis (see Table 3). AI further personalizes discharge instructions to address literacy and language barriers, aiming to lower readmission rates. However, the lack of transparency, potential diagnostic errors, and limitations regarding privacy risks highlight the need for continued clinician oversight.

These applications support pre-hospital first aid by enabling rapid injury detection and vital sign monitoring. The paper’s ED focus highlights a gap in pre-hospital AI solutions, emphasizing the need for bystander-focused interventions. The global burden of injuries underscores the urgency of first aid. WHO reports 4.4 million annual deaths from injuries, including 600,000 from falls, 800,000 from road traffic crashes, and 100,000 from drowning, many preventable with timely first aid (WHO, 2023). Kao et al. (2025) assess YOLO models for detecting chest pain via facial expressions in an ED setting, using a CPR dataset of 1000 patients (1450 pain samples, 1500 no-pain samples).

YOLOv4 and YOLOv6 achieved 80–100% accuracy, compared to 50–70% for YOLOv5 and 35–55% for YOLOv7. YOLOv5, however, provided the fastest training time (3–4 h) and detection speed (0.006 s for YOLOv5n), with a confidence score of 0.746 versus YOLOv7’s 0.597. Its stable loss metrics (decreasing box, objectness, and classification losses) and high precision recall ensure reliability in dynamic settings. YOLOv5n’s low computational cost (1.9 M parameters, 4.5B FLOPs) suits resource-constrained devices for real-time first aid, and the results are summed up in Table 4. Real-world detection results validate its feasibility, though binary classification limits severity assessment (Kao et al., 2025) [13]. YOLOv5’s efficiency supports its use in first aid for real-time pain detection, particularly for cardiac emergencies. The study’s poor performance on the UNBC-McMaster dataset (30–75%) indicates the need for custom pre-hospital datasets.

**Table 3:** Summarizes AI applications from Kachman et al. (2024) and their relevance to first aid

AI Application	Description	Relevance to First Aid
Self-Triage Chatbots	Analyze symptoms for accurate care navigation	Guides untrained responders in assessing injuries
Image Recognition	Detects fractures and hemorrhages in radiographs	Identifies visible injuries (e.g., burns, bleeding)
Vital Sign Monitoring	Predicts deterioration (e.g., eCART for sepsis)	Tracks pulse and respiration in pre-hospital settings
Personalized Instructions	Tailor’s communication for literacy/language	Delivers accessible first-aid guidance for diverse users

**Table 4:** Summarizes YOLO model performance from Kao et al. (2025)

Model	Training Time (hours)	Detection Time (s)	mAP	Confidence Score	Parameters (M)
YOLOv4	≥ 12 (up to 6 days)	Higher than v5	80–82%	-	-
YOLOv5	3–4	0.006	28%	0.746	1.9
YOLOv6	4–5	Higher than v5	-	-	-
YOLOv7	5–7	Higher than v5	-	0.597	-

These studies affirm AI’s role in emergency response. Kachman et al. (2024) highlight AI’s versatility in ED care, while Kao et al. (2025) demonstrate YOLOv5’s suitability for real-time applications. WHO data (2023) emphasizes the need for bystander first aid to reduce preventable deaths, supporting AI-driven pre-hospital solutions [14]. In another study, Cheng (2021) presented the prototype, First-Aid Aider, which combines IoT, AI, and AR to guide novice first responders. By identifying the symptoms with IBM Watson’s IR, providing a secondary diagnosis with Google Assistant’s Dialogflow, and giving first-person first-aid steps with Vuforia’s AR, the prototype was able to identify the injury almost perfectly and give proper visual steps. Although the prototype was tested using 2D images for the target, it struggled with seeing 3D human body parts and there was some room for improvement in the AR, but the significance of this paper exemplified how IoT and AR can be used to alleviate the complexity of first-aid delivery, but gave a transformative way of allowing a uniform and scalable approach to respond to emergencies as untrained bystanders [15].

Liu et al. (2022) proposed an AI-based initiative based on first aid emergency medical knowledge processing, including neural networks, NLP, and 4G/5G for real-time sharing of information of emergency situations between ambulances and hospitals. They used color-coded triage wristbands and diagnosis presented by AI to see the success rates of rescue (e.g., fatalities from hemorrhagic shock, 96.7%), and the triage turnaround time decreased from 19.21 min (*P* < 0.01) to 8.16 min using AI with diagnostic changes, with a 96.35% accuracy. While the AI/ML technology was successful in this first triage emergency event, further development of AI is recommended. In summary, this study demonstrates the benefits of AI in enhancing emergency processes in high-stakes scenarios and provides a scalable model to aid patients in improving outcomes [16].

Senthilkumaran et al. (2023) proposed ARTEMIS, a robot system designed to automate primary triage in mass casualty incidents, utilizing a Unitree Go1 quadruped for locating and assessing victims. The ARTEMIS system used the MediaPipe BlazePose model for human detection and a multi-layer perceptron (MLP) that was trained on the Y-MED-SYN + dataset to perform triage classification, while the communication between the system and operator was implemented using Python’s Speech Recognition and pyttsx3 libraries and delivered over a real-time Graphical User Interface (GUI). The authors acknowledged several limitations with their experiments, primarily English-only communication, a single robot in the first deployment of ARTEMIS, and the lack of real-world assessment, which may limit scalability and adaptability to real MCIs in different contexts. The average triage accuracy achieved by ARTEMIS was 74% and an accuracy of 99% for critical cases, outperforming baseline models such as random forest and Support Vector Machine (SVM), showing significant promise for increasing the efficiency of emergency response [17].

Despite their promising potential, the above works refer to inherent limitations that remain unresolved in actual first aid delivery, including limited 3D spatial recognition, non-portability, external infrastructure dependence, and challenges in adaptability to diverse emergency scenarios. These limitations demand an ultimate solution in the form of a lightweight, real-time, and hands-free AI and AR-enabled system to instruct untrained bystanders in the delivery of effective first aid. In light of this deficit, the proposed AI-supported AR glasses system strives to deliver context-aware, real-time visual and audio first aid guidelines in real-time using embedded edge computing on Jetson Nano, YOLO-based object detection, and speech synthesis, pushing emergency response systems to the next level of scalability and usability in real-world environments.

## Augmented and virtual reality in emergency medical training and first aid

Augmented and virtual reality technologies have emerged as promising tools for enhancing the delivery and retention of first-aid training. Their immersive and interactive nature allows learners to practice critical procedures in realistic yet controlled environments, often without the need for continuous instructor supervision. These technologies are particularly relevant for time-sensitive interventions like CPR and bleeding control, where hands-on skill mastery can significantly influence outcomes. However, despite increasing interest, studies assessing the impact of AR and VR in emergency training have produced mixed findings, with variability in user performance, confidence gains, and long-term retention. The following review examines recent experimental and pilot studies that have implemented AR/VR systems in emergency medical training contexts, identifying both the educational benefits and the technical or methodological challenges that remain.

Aranda-Garcia and colleagues (2023) ran a small pilot study to see how well a short basic life support (BLS) course delivered through smart glasses worked for twelve university students acting in a mock cardiac arrest. Using this hands-free video guidance, the researchers let the students learn, then retest without help while measuring protocol adherence, compression quality, speed, and confidence. After training, the learners performed competently and completed the unassisted test significantly faster (249 s compared with 340 s, *p* = 0.002), even though compression depth stayed slightly below target (4.0 to 4.2 cm). Though limited by the low number of participants and awkward camera angles that hampered exact depth reading, the results mirror European Resuscitation Council advice and support smart glasses as a promising remote tool, especially if paired later with real-time feedback [18].

In 2024, Javaheri and colleagues ran a randomized trial to test RescuAR, an at-home augmented reality app for CPR teaching, working with 43 nursing students and community volunteers. Before the trial, eleven healthcare experts helped shape the app via a design survey; later, testers who trained with RescuAR were compared to a control group that followed standard hands-on classes. The AR group pushed harder and pressed more often, with depths rising sharply (*p* = 0.003) and rates soaring (*p* < 0.0001); overall effective CPR jumped from 23.3 to 61.3% (*p* < 0.0001), while controls finished at 40.4% (*p* = 0.019). Although technical glitches and lab conditions tempered the findings, real-time cues linked to European Resuscitation Council (ERC) standards suggest RescuAR could boost lifesaver training outside formal courses [19].

Sun and colleagues (2024) reviewed and pooled data from nine randomized trials with 855 volunteers to see whether CPR coaching delivered through virtual reality (VR) or augmented reality (AR) was better than standard in-person teaching. Their analysis showed no meaningful gain with VR/AR: chest-compression depth differed by -0.66 mm (95% confidence interval [CI] -6.34 to 5.02; *P* = 0.82), rate by 3.60 compressions per minute (95% CI -1.21 to 8.41; *P* = 0.14), overall performance by -0.05 standard deviations (95% CI -0.93 to 0.83; *P* = 0.91), and the share hitting guideline depth (risk ratio [RR] 0.79; 95% CI 0.53 to 1.18; *P* = 0.26) or rate targets (RR 0.99; 95% CI 0.72 to 1.35; *P* = 0.93). Although the studies varied widely in design and participant skill, the review indicates that headset-based training matches face-to-face practice and could make lifesaving education easier to access; still, future work should harmonize protocols and test skills in emergencies [20].

Birrenbach et al. (2023) completed a prospective feasibility pilot study in Bern, Switzerland, from November 2020 until March 2021, with 45 prehospital emergency physicians and paramedics, to examine a fully immersive virtual reality (VR) simulation for training in Resuscitative Endovascular Balloon Occlusion of the Aorta (REBOA). The study showed that VR training was feasible, with low configuration time, and good usability (SUS median 77.5, IQR 71.3–85), high immersion (Slater-Usoh-Steed median 4.8, IQR 3.8–5.5), acceptable workload (NASA-TLX median 39, IQR 32.8–50.2), and high user satisfaction (USEQ median 26, IQR 23–29). Confidence to perform REBOA significantly improved following VR training (*p* < 0.001), despite the participants having no prior REBOA experience. Although the study was limited in the number of participants and it lacked haptic feedback to better mimic procedural distraction, the VR simulation was an easily scalable, instructor-independent alternative to augment traditional training, with a promise to improve procedural confidence and prevent skills decay, necessitating further research into long-term outcomes [21].

Tharun et al. (2025) evaluated an AR application on Microsoft HoloLens 2 to train 20 non-medical individuals (mean age 27.05 ± 4.2, 8 female) in using a Combat Application Tourniquet for upper limb hemorrhage at the University of Genoa. The AR system, using Unity and Vuforia for QR-code feedback, showed good usability (SUS 75 ± 3.2, *p* = 0.006), positive user experience (UEQ 2.2 ± 0.3, *p* < 0.001), low workload (SIM-TLX significant for task complexity and presence, *p* ≤ 0.05), and minimal simulation sickness (SSQ 0.08 ± 0.07 post-use, *p* > 0.05). Training averaged 240 ± 26 s, with tourniquet placement slowest (96 ± 13 s). Despite engineering backgrounds, familiarization ensured interaction. However, the small sample size and lack of retention or traditional training comparison limit the findings, suggesting that AR’s potential for layperson training is pending further real-world and long-term studies [22].

## Existing first aid technologies and gaps

In the past decade, an increasing variety of digital and mobile technologies have been developed for the facilitation of first aid delivery, especially for minimally trained or untrained bystanders. These vary from mobile applications to intelligent decision-support systems, which aim to improve emergency response speed, accuracy, and confidence. While there have been several studies indicating the capabilities of such technology to enhance some first aid skills like CPR, most of those technologies are still susceptible to practical limitations in usability, real-time response, and response in dynamic or stressful conditions. This section describes some of the most well-known existing first aid technologies, their empirical test performance, and the critical gaps not yet covered, which our proposed AI-driven AR system would attempt to cover.

Metelmann et al. (2021) conducted a randomized comparison study to assess the influence of the smartphone app “HELP Notfall” on the quality of bystander CPR with 200 school pupils in a simulated cardiac arrest scenario. This study was implemented as a case-control study using three groups (control, facultative, and mandatory use of the app). The case-control study used medical simulation with Brayden manikins. Analysis of the data was out via SPSS and Excel. The researchers selected the app for its guideline compliance, and when it was first developed, it was easy to use. “HELP Notfall” enhanced chest compression rate and depth performance metrics. However, there were significant time delays in the commencement of critical actions such as ‘breathing check’ and chest compressions. Limitations of this study were that it was conducted in a simulated environment, the study was limited to adolescent participants, and the 6-week period between training and testing could also reduce the generalizability of the findings. The implications of the study indicate that smartphone apps may contribute to the development of certain CPR metrics; however, the time delays of the app must be considered when being integrated with conventional training methods for bystander CPR to be effective [23].

In parallel with mobile and AR-based interventions for first aid, recent advancements in embedded AI and edge computing have enabled the deployment of lightweight deep learning models on low-power platforms like the NVIDIA Jetson Nano. These developments are particularly relevant to real-time healthcare applications, where latency, portability, and offline processing are critical. Several studies have explored the use of such embedded systems for fatigue monitoring, safety detection, and general object recognition. However, while these systems have demonstrated feasibility in controlled environments, they often fall short in terms of interactive capability, emergency-specific contextual awareness, and multimodal user feedback. The following review discusses recent research on Jetson Nano-based systems, highlighting both their contributions and the limitations that our AIAR First Aid solution seeks to address.

Nicolae, Popescu, and Hossu (2024) used OpenCV and the NVIDIA Jetson Nano to implement a real-time fatigue monitoring system based on facial feature tracking in low-power embedded environments. They showed that lightweight AI can be deployed on edge devices via local video processing and Haar cascade classifiers for detecting fatigue symptoms. Key shortcomings of the system, however, were power and temperature limitations, inconsistency in latency, and intricacies in peripheral integration, like camera synchronization and screen display delays. Their contribution is still restricted in interaction complexity and flexibility, even though it raised significant concerns while deploying edge-based AI. In comparison, our AIAR First Aid system fills in these missing capabilities through a more accurate deep learning model (YOLOv5) for real-time multi-emergency detection (e.g., burns, bleeding, and CPR) and offline text-to-speech for clear directions and readable augmented reality overlays. Sipeed Maixduino Kit is also an alternative hardware platform we are investigating to address the energy and scalability issues with the Jetson Nano. This will make the system cheaper and appropriate for mass deployment in low-resource environments [24].

Nazeer, Qayyum, and Ahad (2022) utilized the NVIDIA Jetson Nano to carry out effective edge-based inference with convolutional neural networks (CNNs), which demonstrated a real-time object detection and identification system. With typical performance metrics of 5–10 frames per second (FPS) for real-time object detection in power-constrained environments, their deployment proved that it is possible to execute machine learning models on embedded devices. Domain-specific metrics, however, like model accuracy or latency in specialized application areas like healthcare, were not presented by the study. Furthermore, its application within time-sensitive, high-risk environments was restricted by the lack of integration with interactive interfaces or multimodal outputs (e.g., auditory feedback). Conversely, our AIAR First Aid employs a light YOLOv5s model that has been trained on emergency-specific datasets (e.g., CPR, burn, and bleeding) and inference-optimized to run about 10 frames per second on edge devices, which is within the capability of Jetson Nano. Additionally, our approach incorporates AR overlays and live text-to-speech guidance, meeting the usability and responsiveness issues raised in Nazeer et al.‘s research [25].

Deng et al. (2023) utilized an improved YOLOv5s model executing on the NVIDIA Jetson Nano, displaying a real-time safety monitoring system for helmet-wearing identification. In their research, they found that lightweight CNN models could be run well on edge hardware with dependable detection accuracy in factory settings and inference rates of about 24 frames per second. The authors demonstrated the viability of the Jetson Nano for low-latency vision-based tasks using hardware-level optimizations, input resolution calibration, and confidence threshold tuning to achieve consistent performance. Their research, however, did not investigate context-aware learning, multimodal feedback, or human-computer interaction—all of which are critical in high-stakes emergency response, even though it focused on binary classification with controlled settings. Our AIAR First Aid app, meanwhile, pushes the capabilities of the Jetson Nano further through the addition of augmented reality overlays and real-time text-to-speech instruction, and multi-class object detection in complicated medical scenarios like bleeding, burns, CPR, and fainting. Untrained individuals can now react appropriately to dynamic situations due to these features. In addition, our approach integrates scenario-based logic flow and interactive support, occupying the gap between visual detection and actual medical intervention, whereas Deng et al. prioritized detection performance. Embedded AI use in public health and emergency preparedness has developed significantly with this shift from passive detection towards active, guided response [26].

## Methodology

**Fig. 2:**
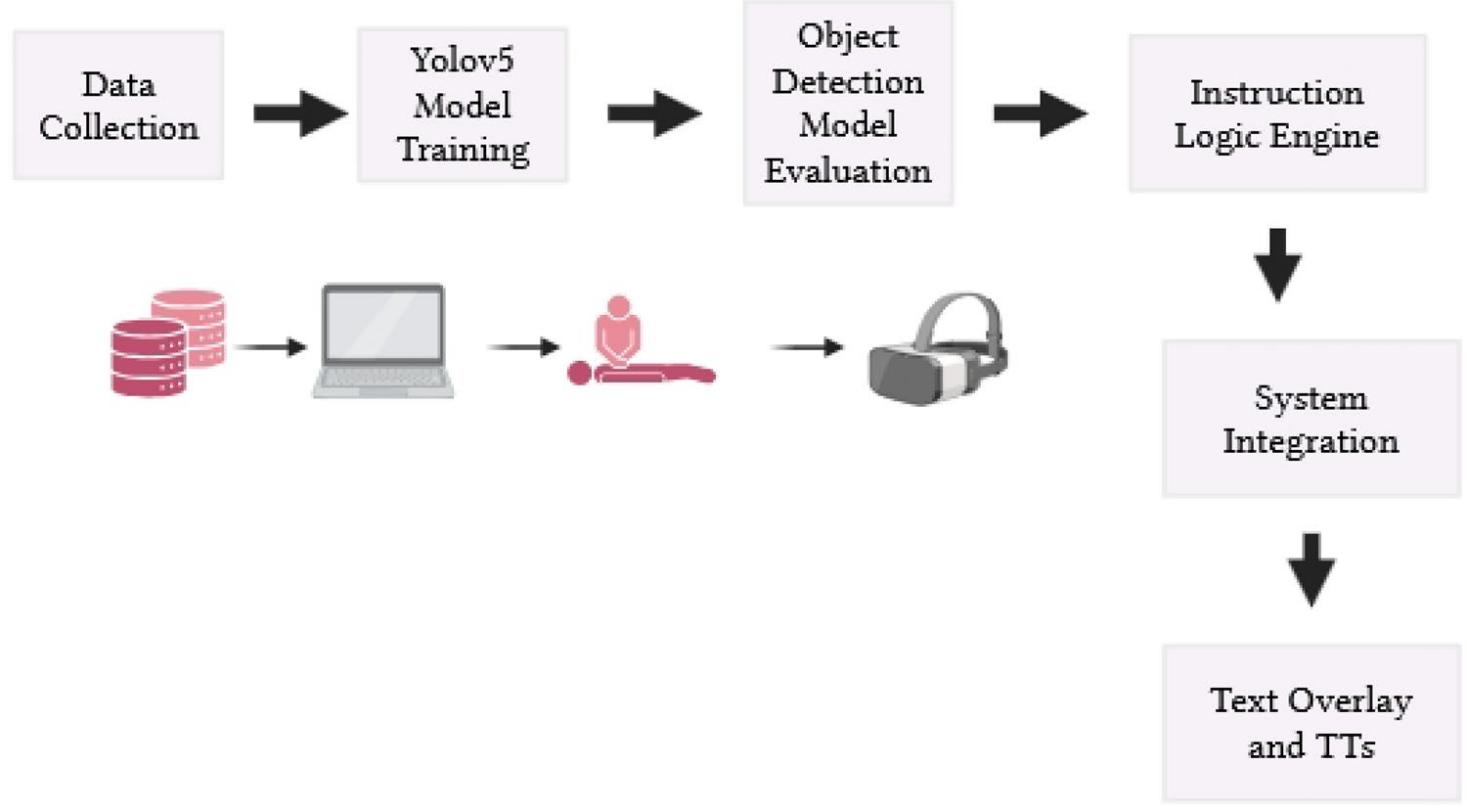
Novel integration of real-time Artificial Intelligence (AI) and Augmented Reality (AR) technologies

This section details the design, development, and implementation of the AIAR system—an AI-based augmented reality solution for first aid guidance. The system integrates the NVIDIA Jetson Nano for real-time processing, a Raspberry Pi Camera for video input, and a TFT LCD mounted on a VR headset for AR visualization. A user-friendly graphical interface, built with Tkinter, prompts users to select an emergency scenario—chest compression, wound, burn, or first aid kit—and provides step-by-step, personalized guidance to assist untrained bystanders until professional help arrives. The development process includes hardware integration, software pipeline construction, decision logic design, and thorough testing to validate system performance. Figure 2 depicts the real-time integration of AI and AR technologies within the system.

### Dataset collection and annotation

The performance and reliability of any deep learning-powered object detection system significantly depend on the quality and diversity of its training data. In the case of the AIAR First Aid initiative, various datasets were gathered, labeled, and preprocessed to support real-time detection of objects connected with emergencies, including chest compression targets, first aid kit contents, bleeding wounds, and burns. These datasets were annotated and sourced primarily using the Roboflow platform, which provided access to a single interface for image upload, annotation, augmentation, and YOLO-compliant exportation.

#### First aid kit component detection dataset

The first dataset [27] comprises basic first-aid supplies such as gauze, bandages, scissors, gloves, and cotton pads. These are necessary for an effective response in the case of emergencies and need to be easily identifiable in the line of sight of the AR glasses. The images were divided into training (513 images), validation (30 images), and test (15 images) sets to maintain a standard 80:10:10 ratio, thereby ensuring sufficient data for model generalization while retaining an independent test set for benchmarking. The images were annotated with bounding boxes representing each of the object classes, and the data was subject to a few augmentations, like changes in brightness and horizontal flipping, to simulate varying environmental conditions.

#### Bleeding detection dataset

Two sub-datasets were gathered to assist in the bleeding detection and severity categorization: one each for evident blood pools and apparent open wounds. The “Wound Dataset” contains 2,295 training images, 420 validation images, and 156 test images representing an exhaustive collection of bleeding scenarios under different lighting and viewpoints. The “Blood Dataset” was smaller with 77 training images, 10 validation images, and 30 test images. However, despite its size, this dataset provided helpful visual cues for detecting surface blood patterns. Both sets were marked to distinguish between light and heavy bleeding and were the basis for scenario-dependent instruction logic. Data augmentation was also performed with rotation, blur, and zoom to increase generalization, especially in the case of low-resolution environments or erratic positions.

#### Burn detection dataset

Another essential function of the AIAR system is the classification of burn severity. For this purpose, a large dataset [28] was collected from Roboflow, which consisted of images classified into three stages of burn as per clinical literature:

- First-degree burns (Class 0): superficial, red, dry without blistering.
- Second-degree burns (Class 1): epidermis and dermis, blistered and painful.
- Third-degree burns (Class 2): reaching the subcutaneous tissues, charred or blackened skin.

The dataset consists of 4,164 train images (88%), 399 validation images (8%), and 200 test images (4%), which is a well-balanced coverage across classes and variations. High-fidelity manual annotation was performed, and contrast normalization, brightness adjustment, and random cropping were performed on the images to train the model under different settings. Class-wise annotation enabled the YOLOv5 model to localize not only the location of the burn but also to predict the severity of the burn, which is essential to generating precise advice for treatment.

#### Chest detection dataset for CPR guidance

For real-time CPR instruction, accurate localization of the chest area is crucial. A specific dataset [29]was prepared and annotated with centering bounding boxes around the anatomical chest region to facilitate hand positioning detection. Seven hundred seventy images were used, consisting of 670 training images, 50 validation images, and 50 test images. The dataset was acquired from clinical images. Augmenting procedures included orientation change, soft blurring, and light variation to simulate real-world variation. The model trained on this dataset shows high reliability in locating the chest area, which is central to the success of CPR overlay reasoning.

### Hardware implementation

**Fig. 3:**
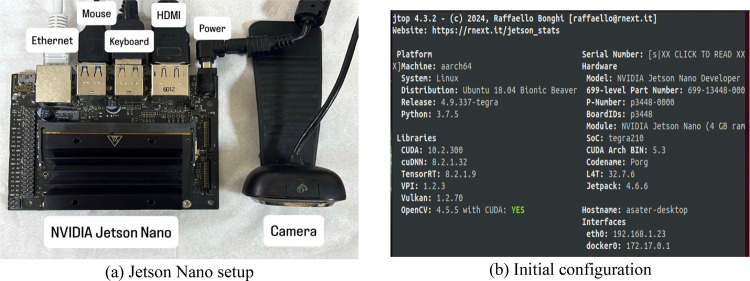
Jetson Nano setup and initial configuration. (**a**) Jetson Nano setup. (**b**) Initial configuration

The proposed system is based on the Jetson Nano board. It is a small, powerful, and affordable single-board computer for AI development, prototyping, and running expansive deep learning frameworks like TensorFlow and PyTorch. Jetson Nano is ideal for developing applications in Robotics, IoT, and edge AI. The operation starts by inserting the microSD card after flashing the JetPack image into the Jetson Nano board. Then connect the monitor, keyboard, mouse, camera, and the Ethernet cable to their corresponding ports on the Jetson Nano board. The Jetson Nano setup and port connections are shown in Fig. 3a. After powering on the Jetson Nano, we checked the initial configuration of the operating system and the installed libraries using the jtop command inside the terminal. Figure 3b shows the installed packages and the Jetpack version on the Jetson Nano.

Creating a virtual environment on the Jetson Nano is an important step. It allows developers to keep Python dependencies and packages separate for each application, avoiding conflicts that could come from incompatible library versions or different dependencies. This is especially important when developing AI and machine learning models for object detection. Large frameworks such as TensorFlow, PyTorch, or OpenCV have specific requirements and/or configurations. The function of each package is explained in Table 5.

**Table 5:** Defining initial packages

Package	Function
JetPack	NVIDIA’s software development kit for Jetson platforms includes OS, libraries, and tools for AI and embedded development.
Python	A high-level, interpreted programming language widely used for AI, scripting, and development on Jetson devices.
aarch64	A 64-bit ARM architecture, the processor type used in the Jetson Nano.
Ubuntu	A popular Linux distribution, with the 18.04 Bionic Beaver version running on the Jetson Nano.
CUDA	NVIDIA’s parallel computing platform and programming model enable GPU-accelerated computing.
OpenCV	An open-source computer vision library, optimized for NVIDIA GPUs with CUDA support.
L4T	Linux4Tegra, NVIDIA’s customized Ubuntu-based OS for Jetson platforms, provides hardware acceleration.

YOLOv5 is a real-time object detection model created by Ultralytics using the PyTorch framework. This model is known to be fast and accurate, providing a single neural network that can predict the bounding boxes and class probabilities directly from full images in a single inference. This offers flexibility between accuracy, speed, and efficiency. Cloning of the YOLOv5 was done with the aid of GitHub. In the context of YOLOv5, a real-time object detection model, with PyTorch as the main coding framework for implementing and training the network, Torchvision provides utilities for data loading and augmentation, for efficient preprocessing of images for detection. Both provide the power for the accuracy and speed of detecting and classifying an object using YOLOv5. We used the official NVIDIA website to guide us in installing PyTorch and Torchvision, which are compatible with both Python 3.8 and the Jetpack. Powering the webcam ensures that the webcam is connected and detected by Jetson. The cheese command was used to open the live feed of the surroundings, and it successfully opened and shows our Jetson connections.

**Fig. 4:**
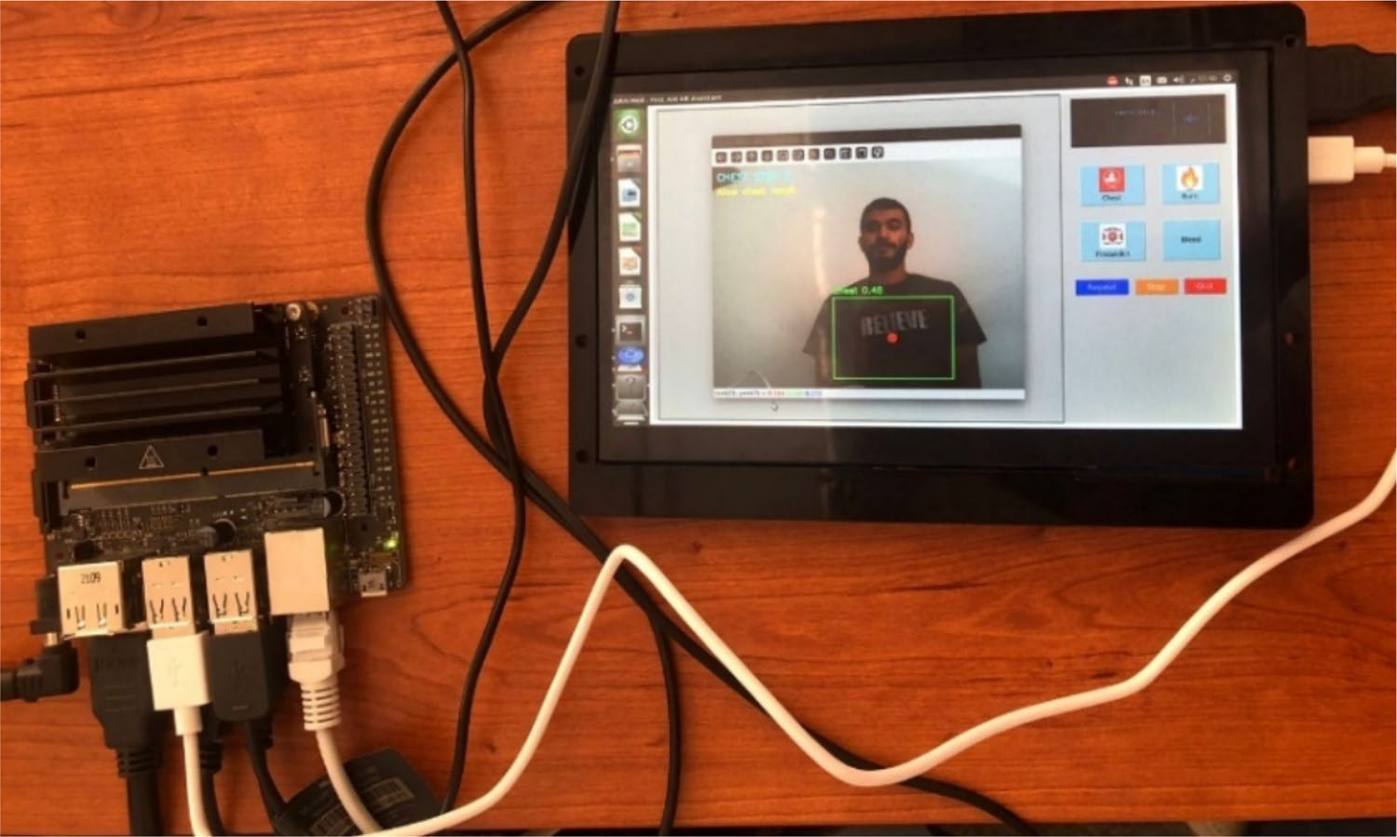
Integration with LCD display for AR simulation

The Jetson Nano, after initializing the object detection system via YOLOv5 and processing real-time input from the connected webcam, outputs the processed frames—including bounding boxes and instructional overlays—through its HDMI port. This HDMI output is directed to a compact LCD screen compatible with the VR box dimensions. The LCD screen is securely mounted inside the VR headset frame, positioned such that the user’s field of view encompasses the full screen. This allows the user to wear the VR headset and experience a live video feed from the webcam, enhanced with overlaid detection information, mimicking an augmented reality environment. Figure 4 illustrates the use of an LCD screen to show the detection output. This approach serves as a cost-effective and accessible prototype for the AI-powered AR system. It bridges the software and hardware components—object detection, real-time video processing, and instructional overlays—with the physical interface that delivers the augmented visuals directly to the user.

### Software architecture

**Fig. 5:**
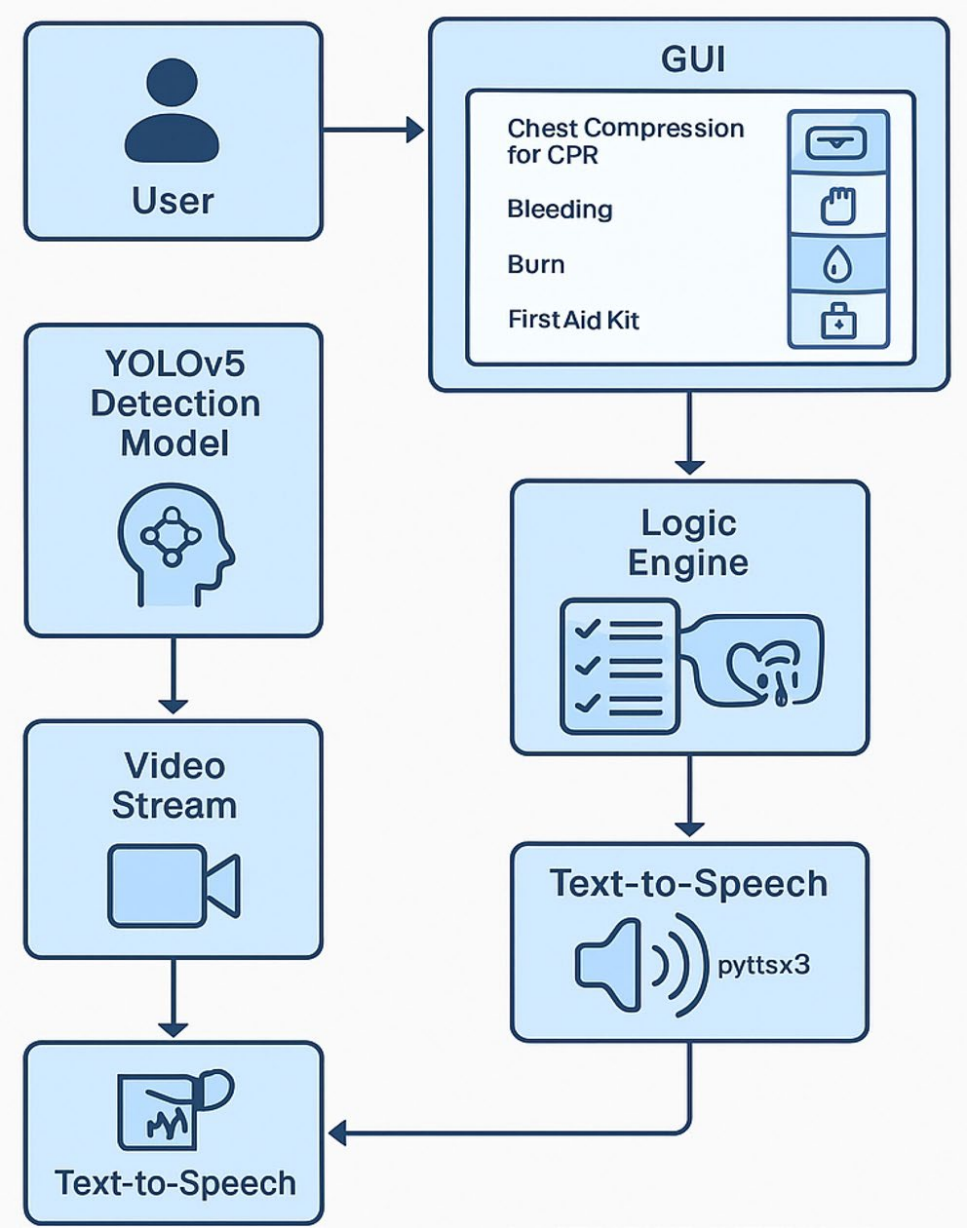
System’s overview 14

The software architecture of the AIAR First Aid system is designed to facilitate real-time, context-aware emergency assistance through a robust integration of computer vision, deep learning, and AR technologies. The core objective is to deliver timely and practical first aid guidance by identifying critical visual cues in the environment, interpreting them based on predefined logic, and providing visual and auditory feedback. This section is structured into four key subsections: YOLOv5 Model Development, Detection Pipeline, Logic Flow and Instruction Mapping, and Framework Integration. Figure 5 shows the flowchart of the developed detection model.

#### The YOLOv5 AI model development

YOLOv5, a state-of-the-art, single-stage object detection model, powers the system’s core object detection functionality. It is known for its exceptional speed and efficiency in real-time applications developed using the PyTorch deep learning framework. YOLOv5 offers a modular design, ease of deployment, and flexibility for fine-tuning specific tasks. The model architecture consists of three main components:

- A CSPDarknet53 backbone, responsible for extracting semantic and spatial features from input images. It uses Cross-Stage Partial Networks (CSP) to enhance learning while minimizing computational complexity.
- A neck implements a Path Aggregation Network (PANet) to improve information flow by merging features at different scales, which enhances detection performance across objects of varying sizes.
- A head performs bounding box regression and classification, predicting object locations and class probabilities in a single forward pass.

YOLOv5 consistently demonstrated superior inference speed compared to previous YOLO versions. For example, Kao et al. (2025) evaluated several YOLO models for detecting facial expressions of chest pain in emergency department settings using a dataset of 1,000 patients (including 1,450 pain and 1,500 no-pain samples). Among all versions, YOLOv5n achieved the fastest detection speed (0.006 s) and shortest training time (3–4 h). It also produced the highest confidence score of 0.746, outperforming YOLOv7’s 0.597, with stable convergence and low computational requirements (1.9 M parameters, 4.5B FLOPs), making it highly suitable for deployment on resource-constrained, real-time first aid systems. While the model’s binary classification setup limited its ability to assess pain severity, its overall performance proved reliable in dynamic environments (Kao et al., 2025) [13].

Additionally, another study [30] was conducted to assess YOLOv5 against Faster R-CNN, a two-stage detector widely used in medical and industrial applications due to its high accuracy. While Faster R-CNN achieved a mean Average Precision (mAP) of 0.90, it suffered from significantly higher inference latency (> 200 ms per frame) on desktop GPUs. It operated at less than 2 FPS on the Jetson Nano. In contrast, YOLOv5s achieved a mAP of 0.84 with only 28 ms inference time on a GPU. They maintained real-time performance on the Jetson Nano, making it far more suitable for low-latency, embedded AR applications.

Given its single-stage architecture, high detection speed, and ease of integration, YOLOv5 was selected as the optimal model for the proposed system. Its compatibility with the Jetson Nano and flexibility for AR overlay make it an ideal choice for enabling real-time first aid assistance in emergency scenarios. Here, we used YOLOv5s, a lightweight variant of YOLOv5, to ensure low latency and compatibility with edge devices like the NVIDIA Jetson Nano. Training was performed using annotated datasets representing various emergency contexts, such as chest compressions, bleeding wounds, burns, and first aid kit components. The datasets were curated through Roboflow and other sources, with data augmentation techniques such as rotation, brightness adjustments, and horizontal flipping applied to enhance generalization.

Model training was conducted with a batch size of 16 over 100 epochs using 640 × 640-pixel resolution. Pre-trained weights were fine-tuned using transfer learning, significantly reducing training time while maintaining high accuracy. The training yielded an average precision (mAP@0.5) of approximately 85% across different classes, making the model suitable for reliable field deployment.

#### Object detection using YOLOv5

In the proposed system, separate YOLOv5 models are trained for each targeted emergency scenario: chest detection for CPR guidance, wound detection for bleeding cases, burn classification, and first aid kit recognition. Each model is trained using transfer learning from pretrained COCO weights and fine-tuned on domain-specific datasets annotated in YOLO format using Roboflow. These datasets contain high-resolution images labeled with bounding boxes around key emergency-relevant items or regions, such as chest areas, bleeding wounds, scissors, or bandages.

Upon execution, the system loads the appropriate detection model based on the scenario selected by the user from the graphical interface. For instance, when CPR is selected, the system invokes the chest detection model to localize the patient’s thoracic region. Detected objects are returned with their respective class labels, confidence scores, and bounding box coordinates. To ensure reliability, detections below a defined confidence threshold (typically 0.25) are filtered out to reduce false positives.

**Fig. 6:**
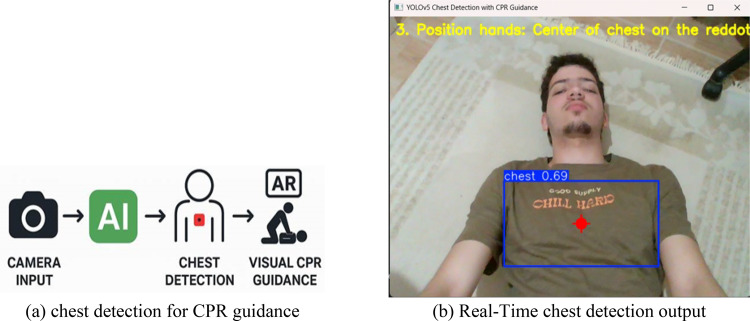
Chest emergency scenario

Figure 6a shows a flowchart of the chest detection and steps followed. The detection results are overlaid on the live camera feed in real time. For chest detection, the system highlights the thoracic region using a green bounding box and displays a red marker at the center of the box, serving as a visual cue for correct hand placement during compressions, as shown in Fig. 6b. The accuracy of this placement is crucial for effective CPR, and visual feedback significantly enhances the user’s confidence and response time.

**Fig. 7:**
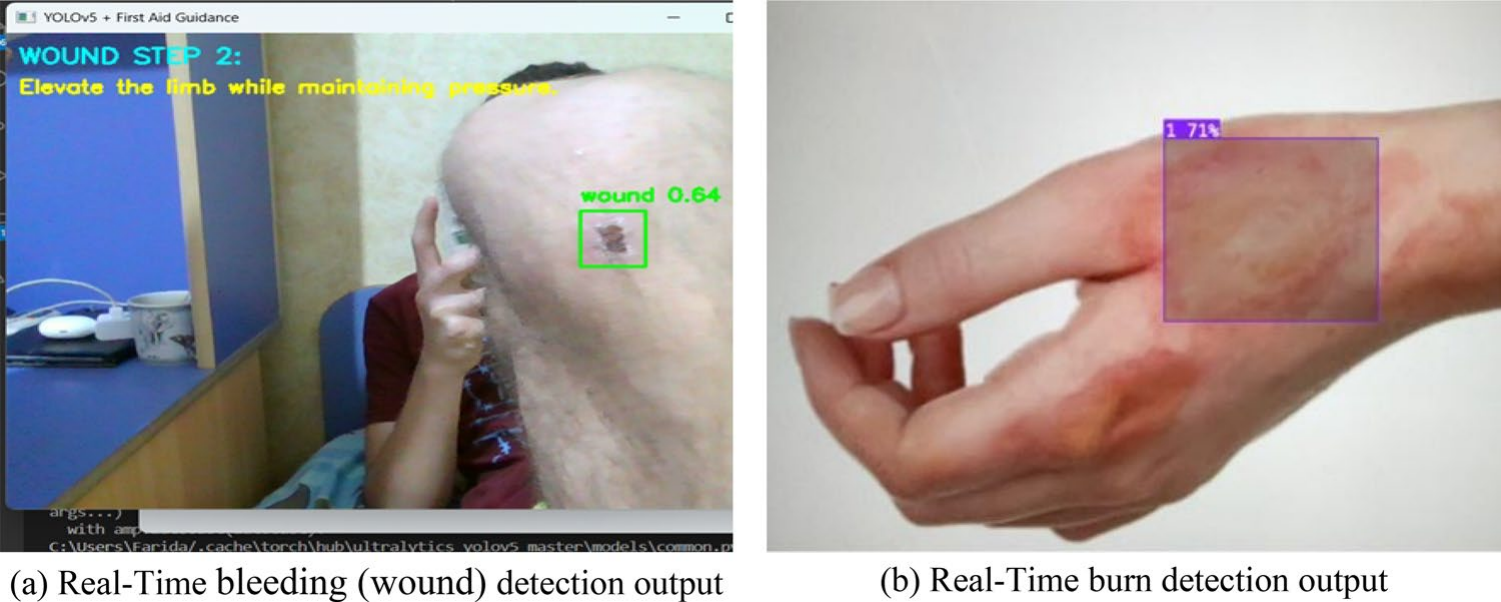
Bleeding (Wound) and burn emergency scenarios

Similarly, for bleeding scenarios, the wound detection model identifies open wounds or bleeding areas, which are highlighted using bounding boxes. Visual overlays provide immediate context to the user, supporting the timely application of pressure or dressing. The bounding boxes dynamically track the wound region across video frames, ensuring robust performance even with slight patient/camera movements. The system adapts to various wound presentations, including clean cuts, lacerations, and hemorrhagic injuries. Figure 7a illustrates model detection in the case of bleeding (wound).

For burn scenarios, the burn classification model identifies and distinguishes between three classes of burns: first-degree, second-degree, and third-degree. Once detected, affected areas are highlighted using bounding boxes, providing clear visual cues to the user. Real-time overlays display the burn class and offer context-aware first aid instructions, such as cooling the area for first-degree burns or avoiding pressure on blistered skin for second-degree burns. The bounding boxes dynamically track the burned regions across video frames, maintaining accuracy even during minor patient or camera movements. The system is trained on a variety of burn appearances, enabling it to adapt to different skin tones, lighting conditions, and burn severity, ensuring a timely and appropriate response. Figure 7b shows model detection output for one burn case.

**Fig. 8:**
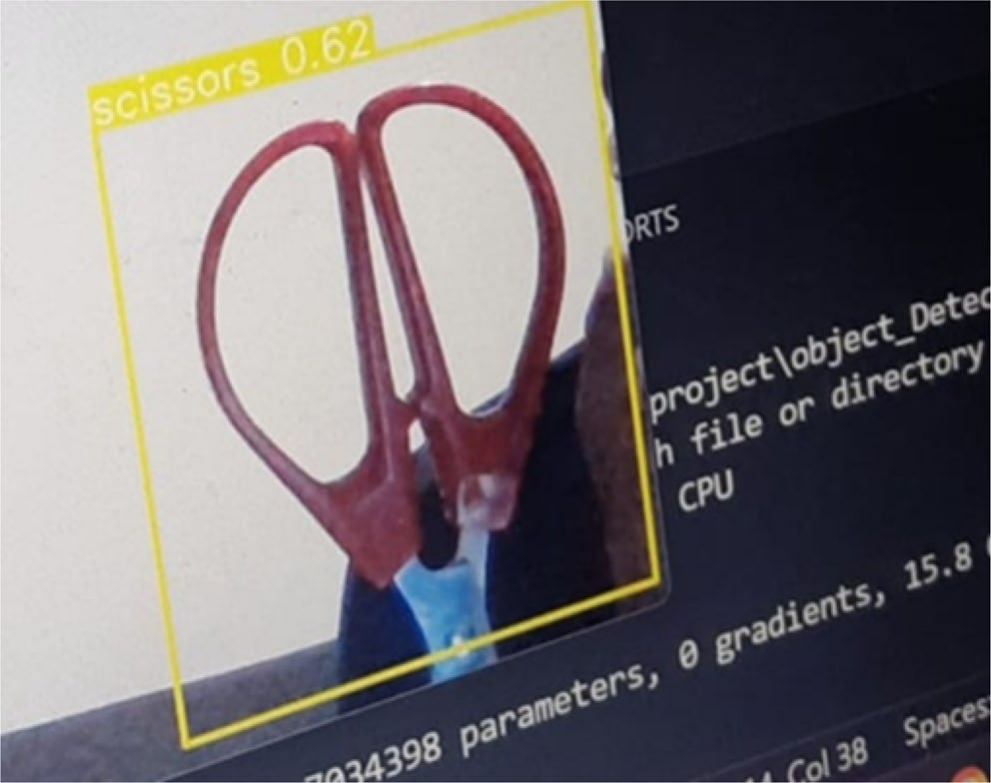
First aid kit detection output (Real-Time Overlay)

In scenarios where the user needs to locate nearby emergency tools, the system activates a dedicated YOLOv5 model for first aid kit detection. This model is trained to recognize standardized first aid kit containers in diverse settings, including indoor, outdoor, and cluttered environments. Once detected, the system highlights the kit’s location on the video stream and may optionally provide audio or visual prompts directing the user toward it. This functionality is particularly critical in panic-prone situations where quick access to medical supplies can save lives. By accurately identifying critical visual cues in emergency scenarios, the YOLOv5 detection module is the foundational layer upon which all decision logic, instructional guidance, and augmented reality overlays are built. The model was able to detect equipment in the first aid kit and was able to define it as shown in Fig. 8.

#### Logic flow and instruction mapping

The logic engine governs the system’s ability to translate visual detections into meaningful first-aid instructions. A scenario-specific instruction map is maintained using Python dictionaries, linking object classes to procedural steps. This mapping provides the foundation for dynamic, context-sensitive guidance in emergency scenarios. The instruction flow is aligned with standardized first-aid protocols and best practices issued by health organizations. For example, in a chest compression scenario, the system initiates a sequential CPR guide once the chest is accurately detected. Instructions include checking responsiveness, calling emergency services, initiating compressions, and monitoring breathing. Each instruction step is timed to appear every five seconds and is reinforced through synchronized audio output using a text-to-speech engine.

In bleeding scenarios, the system’s logic distinguishes between minor and severe wounds based on object size and the bounding box of confidence. A “severe bleeding” subroutine is activated if a large wound is detected. This initiates a cascade of steps: apply direct pressure, elevate the affected limb, and seek immediate professional help. The user is guided visually and audibly through each action. For burn injuries, the detection output is classified into severity levels first, second, or third degree based on color, area, and texture cues. Each burn class triggers a distinct instructional flow. For example, minor burns prompt instructions to cool the burn under water, while third-degree burns instruct users to avoid applying creams and cover the area with a sterile cloth until help arrives.

#### Framework integration

The AIAR First Aid system leverages a modular software architecture developed entirely in Python, integrating a suite of libraries to deliver real-time, scenario-specific guidance. The framework combines PyTorch for deep learning inference, OpenCV for detection visualization, PyQt5 for the graphical user interface (GUI), and pyttsx3 for offline text-to-speech synthesis. Each component is designed to function cohesively, enabling the system to provide immediate, context-aware first aid instructions in a user-friendly format.

Upon startup, the user is presented with a full-screen GUI that acts as the central control center. The interface displays a clear visual layout with selectable emergency scenarios represented by labeled icons, including chest compressions, bleeding, burns, and locating a first aid kit. Once the user selects a scenario, the system prompts for the desired number of instruction repetitions, enhancing flexibility in time-critical situations. A subprocess is then launched to execute the corresponding detection script, which activates the appropriate YOLOv5 model and procedural logic. The detection output, including bounding boxes and AR-based instruction overlays, is rendered in a separate OpenCV window. Simultaneously, the system delivers spoken instructions via the pyttsx3 library to ensure auditory reinforcement. These instructions are managed through a background thread using a Python Queue to maintain non-blocking performance. Speech events are intelligently managed to prevent redundancy by comparing each instruction against a cache of previously spoken steps. The system maintains a consistent speech rate of approximately 170 words per minute and introduces minimal latency, typically under 200 milliseconds, ensuring smooth and responsive guidance.

Multithreading is employed to handle parallel operations such as instruction dispatching and speech synthesis, ensuring that the GUI remains responsive during model execution. The clean separation between interface and model logic allows for robust error handling and system stability, even in challenging or unpredictable environments. This architecture not only supports real-time detection and guidance but also lays the foundation for future enhancements such as multilingual support, voice interaction, and embedded video feed integration. The result is a cohesive and intelligent AR-based assistance platform tailored to enhance situational awareness and empower users in emergency response scenarios.

**Fig. 9:**
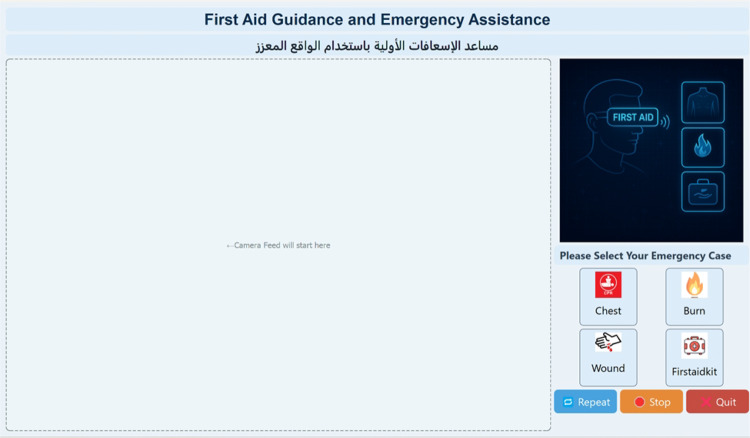
The complete developed system GUI

The graphical user interface (GUI) of the AIAR system is designed with clarity, responsiveness, and usability as primary goals, as shown in Fig. 3.20. Built using PyQt5, the GUI occupies the full screen and is structured to guide the user through the emergency assistance workflow with minimal cognitive load. The interface is visually divided into two main sections: a large, left-aligned placeholder where the real-time detection feed can be displayed or dragged in, and a right-aligned control panel. The control panel includes a high-resolution banner, a descriptive instruction label, and clearly labeled icons representing distinct emergency scenarios such as chest compression, burn injuries, bleeding, and locating a first aid kit. Each icon is styled with consistent dimensions and modern flat design, promoting visual uniformity and ease of interaction. Figure 9 shows the complete developed system GUI.

User input is primarily handled through buttons and dialog prompts. When a model icon is selected, the GUI initiates a prompt asking how many times the user wishes to repeat the associated first aid instructions. This step ensures adaptability to user preferences and situational urgency. Beneath the emergency icons, three control buttons - “Repeat,” “Stop,” and “Quit” - provide users with clear command options to manage ongoing processes. The entire interface is designed to scale across resolutions and devices, with dynamic layouts ensuring that elements remain aligned and legible regardless of screen size. Background colors, font choices, and visual elements are selected to maintain a balance between aesthetic appeal and accessibility, ensuring that the interface remains intuitive even under stressful conditions.

## Results and discussion

This section discusses the results of the AIAR system designed to guide laypersons through first aid procedures. The Tkinter-based welcome interface ensures user-friendly scenario selection, while the YOLOv5 model, text-to-speech integration, and real-time AR overlays deliver intuitive, scenario-specific guidance. The system’s robust hardware and software integration support its goal of providing practical emergency assistance. The section also discusses the detailed testing results and user feedback.

### Real-time detection of emergency scenarios

**Fig. 10:**
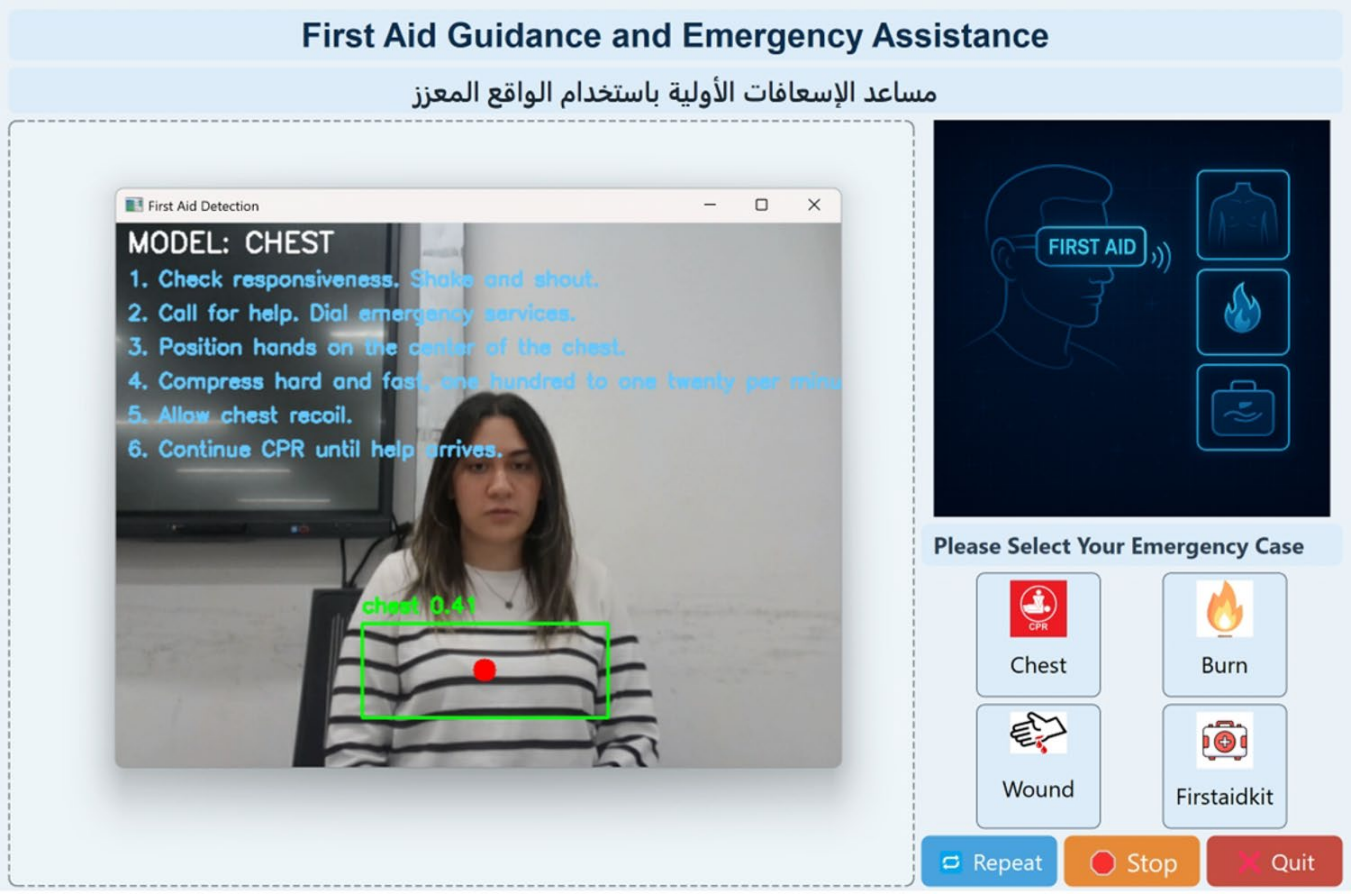
Real-time chest detection using the developed GUI

Figure 10 shows the output of the chest detection model within the developed AI-powered First Aid Guidance system. The interface captures a live video feed from the camera, over which a deep learning model, trained using YOLOv5, performs real-time object detection. In this instance, the system identifies the “chest” region, which is crucial for initiating CPR in unresponsive individuals. The detected area is highlighted with a bounding box and a center dot, helping users quickly locate the appropriate region for compression. Once detection is successful, the system activates the corresponding logic flow and begins presenting step-by-step CPR instructions. These instructions are stacked on the left side of the screen in chronological order, each rendered in a readable font. This design ensures that users can follow along visually without losing track of prior steps. Additionally, the instruction overlay is synchronized with a text-to-speech (TTS) engine, allowing for simultaneous auditory guidance. This dual-modality design supports users who may be operating under stress or who have limited literacy. The overall figure demonstrates the system’s ability to combine computer vision and procedural logic to deliver real-time, context-aware first aid support for cardiac emergencies.

**Fig. 11:**
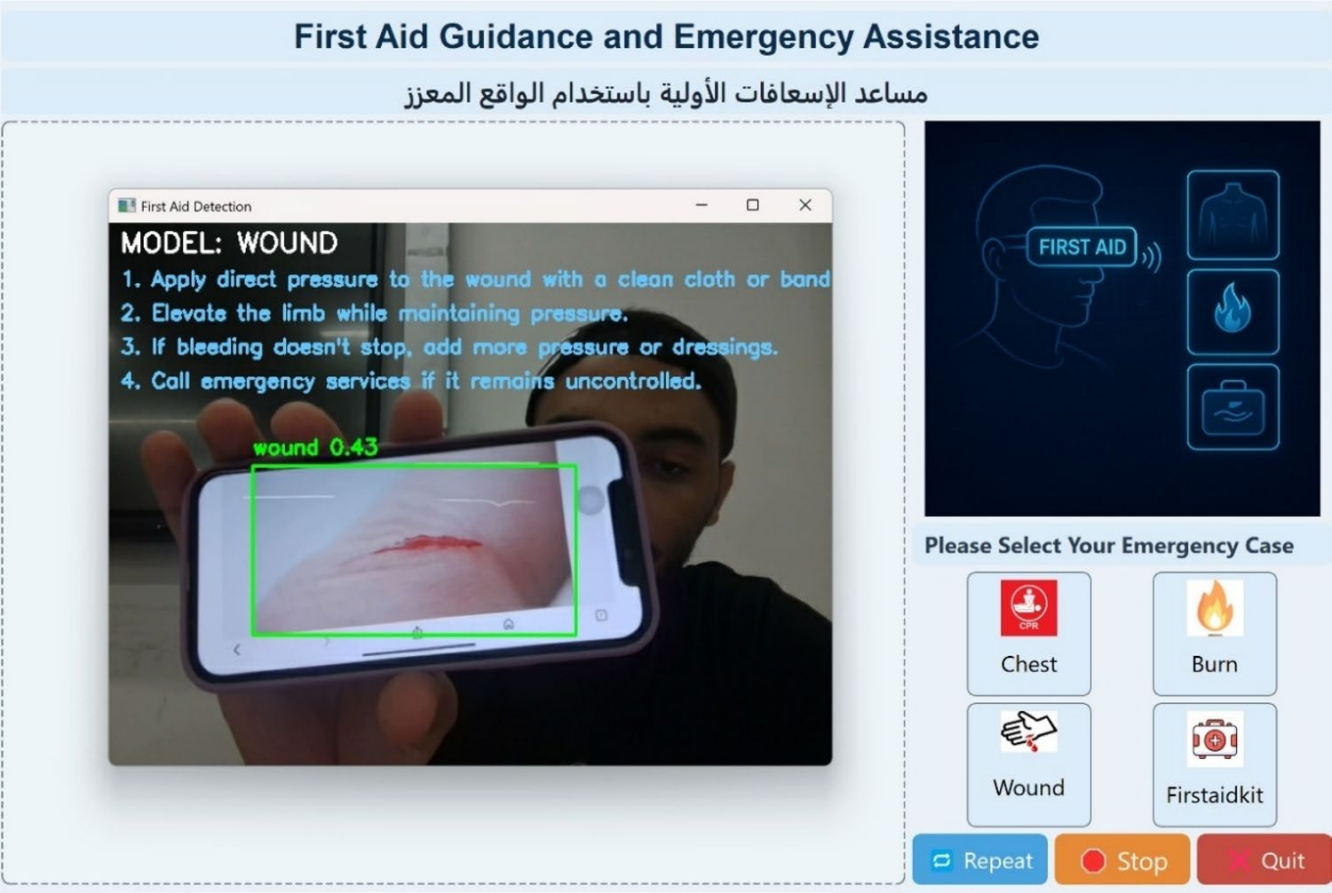
Real-time wound detection using the developed GUI

Figure 11 illustrates the emergency detection system’s output in a bleeding scenario. The wound detection model identifies visible bleeding areas in real time. Detected wounds are enclosed with bounding boxes, allowing users to pinpoint the injury location instantly. In addition to the visual cue, the system provides step-by-step on-screen and voice-guided instructions to help the user apply appropriate first aid, such as applying direct pressure, elevating the limb, and monitoring the person’s condition.

**Fig. 12:**
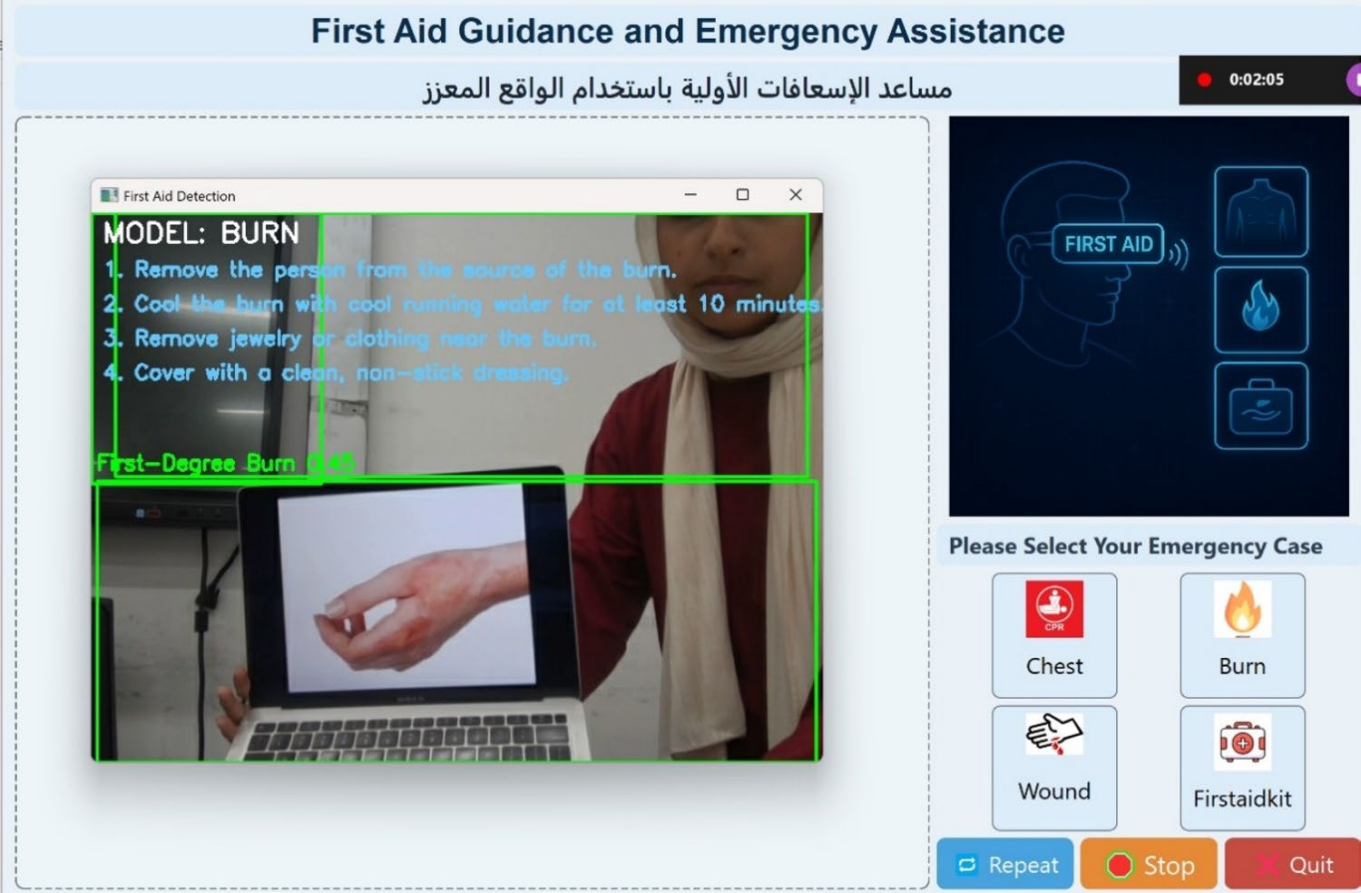
Real-time burn detection using the developed GUI

Figure 12 demonstrates the system’s performance in a burn-related emergency. The burn detection model identifies the affected area and overlays a bounding box along with contextual instructions tailored to the burn’s severity, such as cooling the area under running water and covering it with a non-stick dressing. The model operates in real time, maintaining accuracy despite movement, and ensures that even users without a medical background can respond quickly and effectively.

### Testing and validation

Testing and validation were developed and implemented to ensure the reliability, accuracy, and usability of the AIAR First Aid system. The evaluation is performed for object detection, instructional mapping, interface responsiveness, and speech synchronization, each targeting a specific component of the software architecture.

#### Detection model evaluation

**Fig. 13:**
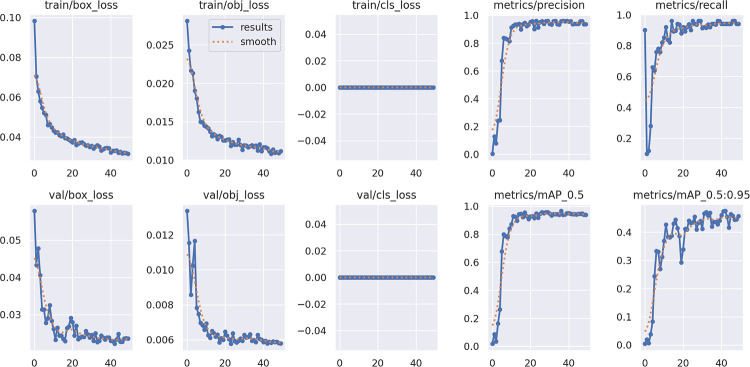
Chest’s training, validation loss curves, including precision, recall, and mAP performance metrics

**Fig. 14:**
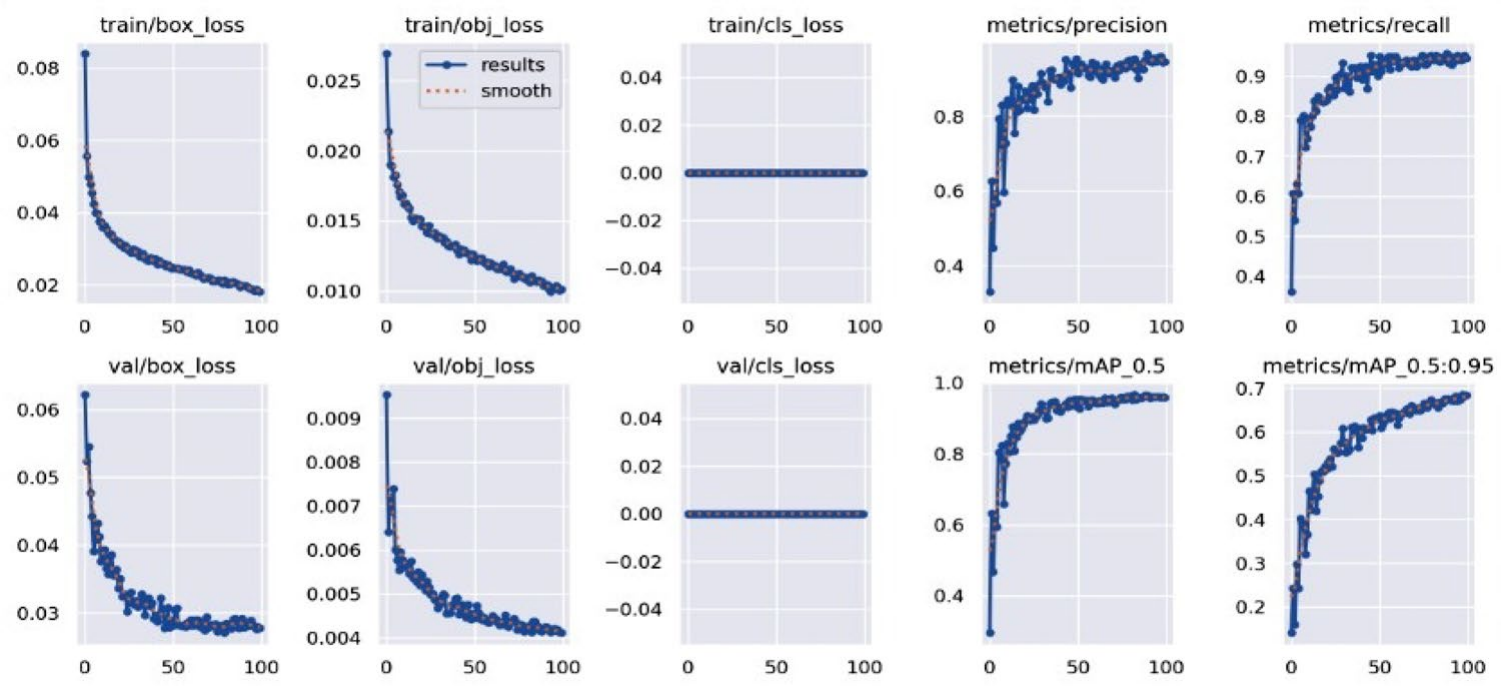
bleeding’s training, validation loss curves, including precision, recall, and mAP performance metrics

**Fig. 15:**
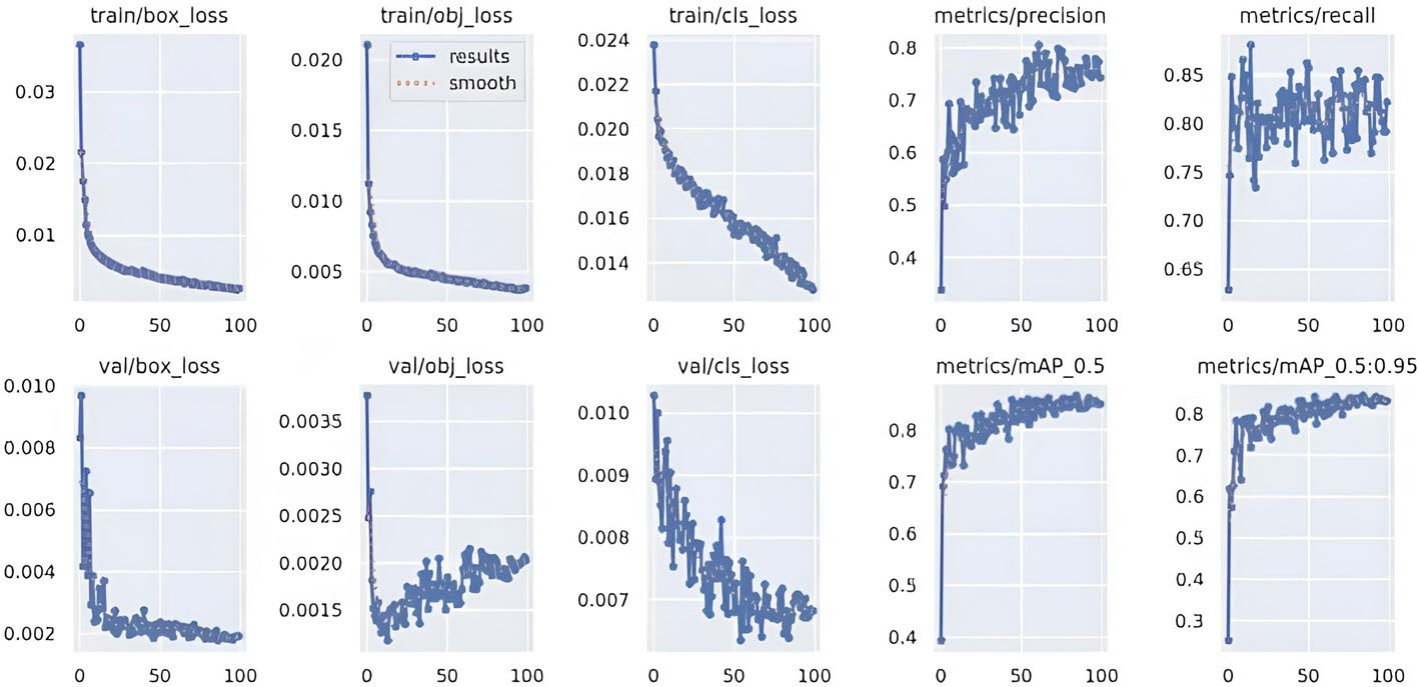
Burn’s training, validation loss curves, including precision, recall, and mAP performance metrics

**Fig. 16:**
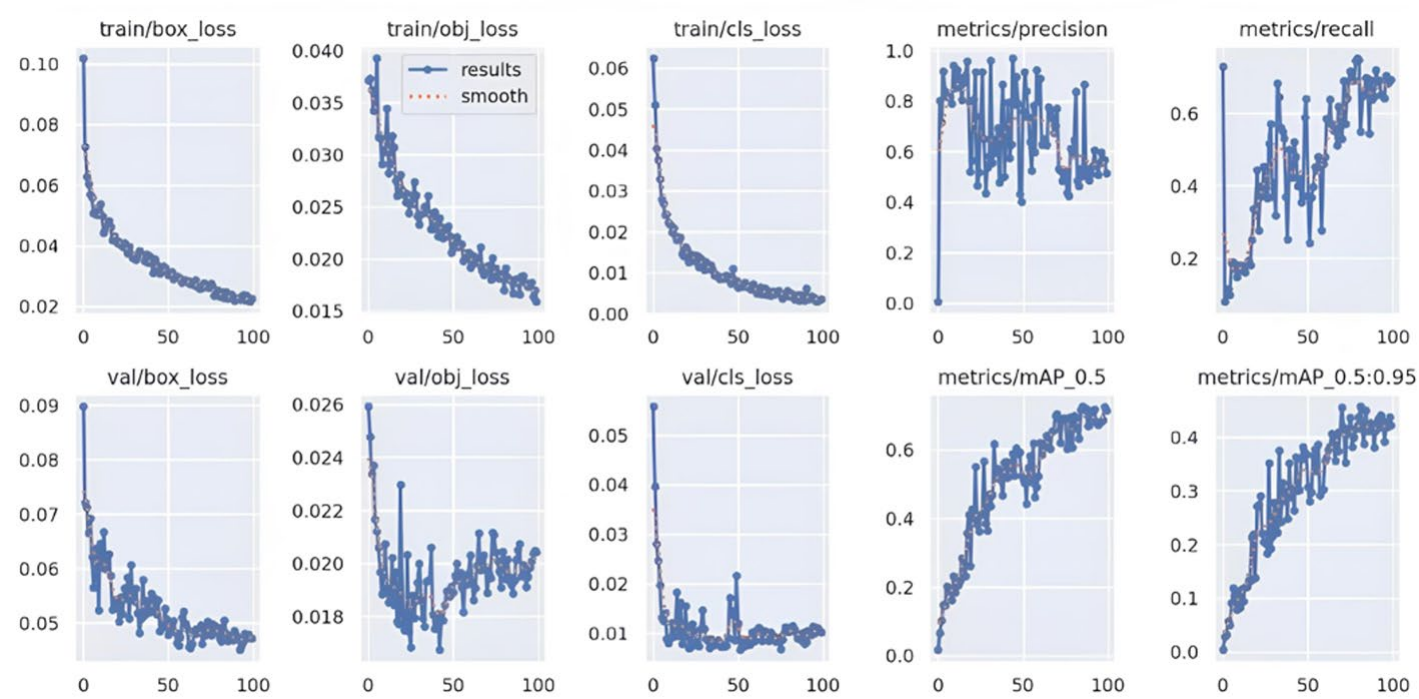
First Aid’s training, validation loss curves, including precision, recall, and mAP performance metrics

The YOLOv5-based object detection models were assessed using standard performance metrics, including precision, recall, and mean Average Precision (mAP). Custom datasets for chest regions, wounds, bleeding, burns, and first aid kits were split into training, validation, and testing subsets using an 80:10:10 ratio. The model was trained for 100 epochs with a batch size of 16 and image resolution set at 640 × 640 pixels. Validation was performed on an independent dataset containing real-world emergency images that were not used during training. Figures 13, 14, 15 and 16 represent training and validation metrics for the four models trained on different datasets, for a detection or classification task. The train/box_loss, train/obj_loss, and train/cls_loss curves show the decline in box, objectness, and classification losses during training, indicating the model is learning to accurately localize and classify dataset-related features, with smoothed trends confirming stable convergence. Similarly, val/box_loss, val/obj_loss, and val/cls_loss reflect consistent loss reduction on the validation set, suggesting good generalization. The metrics/precision and metrics/recall curves demonstrate increasing precision and recall over epochs, indicating improved accuracy and the ability to detect true positives. The metrics/mAP_0.5 and metrics/mAP_0.5:0.95 curves, showing mean average precision at Intersection over Union (IoU) thresholds of 0.5 and 0.5 to 0.95, respectively, rise steadily, reflecting robust overall performance across different detection thresholds on the different datasets. These plots jointly provide insights into the training process, helping to identify overfitting, underfitting, or convergence issues, and guide hyperparameter tuning or model adjustments for optimal performance on the burn dataset. The boxes’ representation and importance are given in Table 6.

**Fig. 17:**
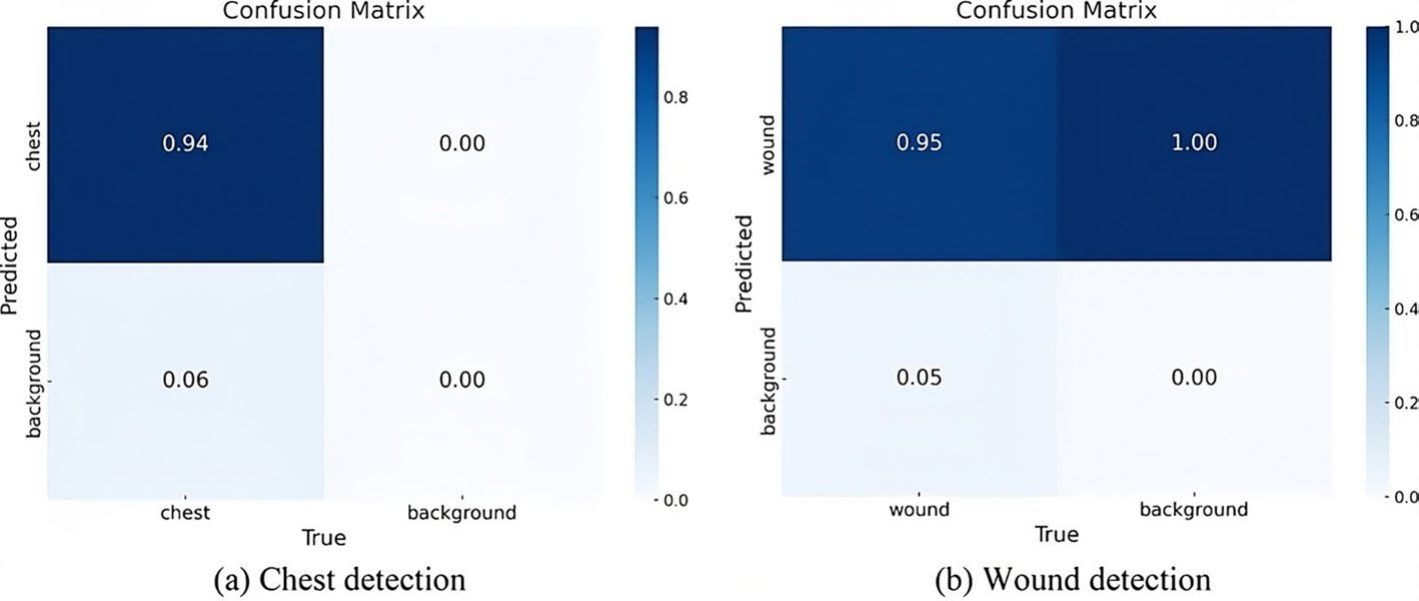
Confusion matrices of chest and wound detection

A confusion matrix is another performance metric used to evaluate the models. Figure 17a shows the performance of a classification model predicting ‘chest’ and ‘background’ classes. The confusion matrix for the chest dataset shows the following values:

- 94% of actual “chest” cases were correctly predicted as “chest”, and 0% cases were incorrectly predicted as “chest” (background predicted as chest).
- 6% of actual “chest” cases were incorrectly predicted as “background”.
- 0% cases were correctly predicted as background, likely due to an imbalanced dataset or lack of “background” samples, as this model just detects chest, and doesn’t focus on “background”.

Figure 17b shows that the high true positive rate (0.95) indicates robust detection of “wound” instances, aligning with the model’s training objective. However, the 1.00 false positive rate for “background” stems from the dataset’s labeling strategy, where only “wound” regions were annotated. In YOLOv5, unlabeled regions are typically treated as negative samples or ignored during training, but the model may still assign “wound” labels to “background” areas due to the absence of explicit “background” annotations. This behavior is expected and does not necessarily indicate a model flaw but rather a limitation of the dataset. The 0.05 false negative rate suggests minor misclassifications of “wound” instances, possibly due to ambiguous boundaries or insufficient training data.

**Table 6:** The boxes’ representation and importance

The Plot	Representation	Importance
train/box_loss	Represents the error in predicting the bounding box coordinates (e.g., position and size) during training.	Explain how well the model learns to localize objects, with a decreasing trend indicating improved spatial accuracy.
train/obj_loss	Measures the error in objectness score prediction (likelihood of an object being present) during training.	Correctly identifies the presence of burn-related features, with a decline showing better object detection confidence.
train/cls_loss	Indicates the error in classifying the object type during training.	Assess classification accuracy, with a downward trend reflecting improved category prediction.
val/box_loss	Shows the bounding box error on the validation set.	Evaluates the model’s generalization to unseen data, with a decreasing trend suggesting robust localization.
val/obj_loss	Represents the objectness error on the validation set.	Tracking confirms the model’s ability to generalize object presence detection, with a decline indicating consistent performance.
val/cls_loss	Measures the classification error on the validation set.	Assess how well the model generalizes its classification capabilities, with a decreasing trend showing reliable category prediction.
metrics/precision	Displays the ratio of correctly predicted positive instances to total predicted positives.	Evaluates the accuracy of positive predictions, with an increasing trend indicating fewer false positives.
metrics/recall	Shows the ratio of correctly predicted positive instances to all actual positives.	Assess the model’s ability to detect all relevant burn instances, with a rising trend indicating better sensitivity.
metrics/mAP_0.5	Represents the mean average precision at an IoU threshold of 0.5, summarizing detection quality across different recall levels.	Estimates the overall performance at a standard threshold, with an increase reflecting improved detection accuracy.
metrics/mAP_0.5:0.95	Indicates the mean average precision averaged over IoU thresholds from 0.5 to 0.95.	Evaluates the detection performance across a range of thresholds, with a rising trend showing robust and precise localization.

#### User interface and interaction testing

The GUI’s usability and responsiveness were tested through user interaction studies involving participants with no formal medical training. Participants were instructed to perform simulated first aid scenarios using the dropdown menu and respond to onscreen and spoken instructions. The interface was evaluated using the System Usability Scale (SUS) based on task completion rate, error frequency, and user satisfaction.

#### Text-to-speech synchronization

The synchronization between visual instructions and auditory cues was evaluated through time-stamped logging during execution. Any delays or overlaps were recorded and analyzed to ensure temporal coherence. The system maintained a maximum asynchrony of ± 250 milliseconds, within acceptable limits for multimodal guidance systems.

## Limitations and challenges

While the AIAR First Aid system demonstrates promising potential in merging AI and AR for real-time emergency support, several technical and practical limitations affect its current implementation. Hardware constraints are among the most pressing issues, with the NVIDIA Jetson Nano’s limited processing power causing occasional inference delays, especially when handling high-resolution inputs or multiple detections simultaneously. The bulky form factor of the VR headset, assembled from off-the-shelf components, reduces user comfort and may interfere with usability during high-stress scenarios.

Furthermore, the system’s dependence on external battery packs limits its operational duration and mobility, making it less viable for long-term field deployment. Detection accuracy is also impacted by factors such as small or imbalanced datasets, particularly for bleeding and chest detection, and environmental conditions like poor lighting or motion blur. Additionally, the system currently supports only four emergency scenarios, leaving out other common conditions such as fractures or choking.

On the usability and deployment side, the system faces challenges related to interface design and accessibility. While a GUI with scenario selection is available, it may be unintuitive for users under stress or with limited digital experience. Instructions delivered via text-to-speech improve accessibility but may lack clarity or be poorly paced for users with low health literacy or attention span in emergencies. The lack of haptic feedback further limits sensory support in noisy environments. Deployment at scale also presents obstacles, including high prototype costs, limited availability in low-resource settings, and varying levels of digital literacy among target users. Although the system functions offline, it lacks localization features, and infrastructure gaps could restrict future reliance on network connectivity. Lastly, ethical and regulatory concerns must be addressed. The system’s processing of live video raises data privacy issues, and its lack of formal clinical certification or legal frameworks for AI-assisted intervention introduces risks related to medical liability and user accountability.

## Future work

The AIAR First Aid system has laid the foundation for an innovative, accessible, and AI-powered augmented reality platform that assists non-experts in delivering emergency care. However, realizing its full potential as a scalable, field-deployable, and context-aware system requires deliberate enhancements across hardware and software domains. Future work will pursue multiple parallel directions, focusing on affordability, form factor miniaturization, contextual intelligence, and immersive AR interaction. In terms of hardware development, a key future direction is toward creating affordable, scalable edge computing solutions. Current implementations rely on relatively high-end embedded platforms, which may hinder widespread adoption in low-resource or remote environments. Transitioning to low-cost, energy-efficient platforms such as the Sipeed Maixduino Kit based on the RISC-V Kendryte K210 chip represents one promising approach [31]. This shift involves optimizing detection models for low-memory environments, leveraging hardware acceleration features, and porting user interfaces to lightweight frameworks, which together will make the system more portable, power-efficient, and accessible for mass deployment.

Another crucial avenue is the evolution of AIAR into a wearable device, specifically through the development of lightweight, intelligent AR glasses. These smart glasses would incorporate integrated AI chips, onboard cameras, and waveguide displays to provide system guidance into the user’s field of view. Such a form factor would facilitate hands-free operation, enabling untrained bystanders to respond swiftly and effectively in emergencies without the need for external devices. Incorporating advanced edge AI hardware like the Qualcomm Snapdragon XR chips would allow for real-time inference, gesture recognition, and spatial audio feedback, further enhancing usability in high-stress situations. Furthermore, advancing beyond simple linear logic flows, future iterations of AIAR will incorporate more sophisticated, context-aware, and adaptive interaction capabilities. This involves integrating pose estimation, environmental mapping (SLAM), and depth sensing to tailor guidance dynamically based on user responses and environmental cues. Such improvements would enable the system to provide personalized corrections—e.g., adjusting CPR rhythm or hand placement—mimicking expert human feedback. Additionally, implementing natural language dialogue interfaces would allow users to communicate verbally with the system for clarification or additional instructions, thus improving usability amidst noisy or chaotic conditions. Enhancing detection robustness and accuracy will also be a focus, with plans to incorporate advanced models trained on diverse, domain-specific datasets to improve reliability under various lighting, occlusion, and perspective conditions. In addition, rigorous testing through clinical trials and cross-sector deployments will be essential to validate AIAR’s effectiveness and safety in real-world scenarios. Collaborations with medical institutions, emergency responders, and disaster relief agencies will facilitate comprehensive simulation trials to evaluate usability, instruction accuracy, latency, and intervention success rates. Insights gained will inform necessary regulatory approvals and popularize the system across healthcare, military, and public safety domains, paving the way for widespread adoption of AI-assisted emergency response solutions.

The proposed AR-powered first aid system holds substantial promise for diverse real-world applications beyond emergency medical response. Its capacity to deliver real-time, context-aware guidance empowers untrained bystanders to effectively assist in critical situations such as cardiac arrests, bleeding, burns, and fainting episodes. By enabling immediate intervention, the system can substantially improve patient outcomes and survival rates, particularly in out-of-hospital and remote settings where timely professional medical assistance may be delayed. In addition to emergency response, this technology offers valuable educational and training opportunities. Medical students and first responders can utilize it as a simulation tool to practice and refine intervention skills in a controlled, realistic environment. The integration of multimodal guidance—visual AR overlays, text instructions, and speech synthesis—ensures accessibility across varying literacy levels and stress conditions, fostering confidence and competence in emergency scenarios.

Furthermore, the development of wearable AR devices, such as smart glasses with integrated AI chips and environmental sensing capabilities, enhances portability and user-friendliness. These devices could facilitate continuous real-time monitoring and rapid response, not only in healthcare but also in military, disaster relief, and industrial safety contexts. Their hands-free operation allows untrained personnel, volunteers, or field workers to deliver critical aid swiftly, reducing the dependency on specialized training. Advancing hardware solutions with low-cost, energy-efficient platforms broadens deployment potential, making the system accessible in low-resource and underserved regions. Future integration of environmental mapping (SLAM), pose estimation, and natural language interaction will further personalize guidance, improve robustness under diverse conditions, and streamline user experience. By bridging the gap between detection and intervention, this system exemplifies a significant step toward democratizing emergency healthcare, extending life-saving capabilities to a broader audience, and ultimately contributing to more resilient and responsive communities.

## Conclusion

This paper introduces a novel AI-powered augmented reality (AR) system designed to assist untrained individuals in delivering effective emergency first aid. By integrating real-time object detection through YOLOv5, edge computing on the Jetson Nano platform, and multimodal guidance via AR overlays and text-to-speech, the system provides context-aware, hands-free instructions tailored to critical scenarios such as bleeding, burns, fainting, and CPR. The development of domain-specific datasets and medically validated decision flows ensures reliability and relevance in emergencies. Experimental results demonstrate that the proposed system offers high usability, responsiveness, and adaptability, significantly bridging the gap between detection and actual intervention in out-of-hospital environments. Despite these promising outcomes, limitations remain concerning hardware constraints, model accuracy under diverse real-world conditions, and energy consumption, which may affect deployment scalability. Future directions include the development of more compact and energy-efficient AI hardware, improved robustness in variable environments, and comprehensive clinical validation to ensure effectiveness and safety. Eventually, this work advances the field of emergency healthcare by enabling rapid, accessible, and guided responses, empowering untrained bystanders to act confidently and potentially save lives in critical situations.

## Data Availability

No datasets were generated or analysed during the current study.
